# Engineered Outer Membrane Vesicles for Antigen Delivery: Exploratory Study on Adjuvant Activity and Systemic Reactogenicity

**DOI:** 10.3390/vaccines13060552

**Published:** 2025-05-22

**Authors:** Lu Lu, Lina Zhai, Qikun Ou, Shuli Sang, Chen Cao, Yiyan Guan, Yunyun Mao, Yanfang Zhai, Kai Li, Rui Yu, Yanchun Wang

**Affiliations:** 1 Laboratory of Advanced Biotechnology, Beijing Institute of Biotechnology, 20 Dongda Street, Beijing 100071, China; lulu52102022@163.com (L.L.); sangshuli@bmi.ac.cn (S.S.); 1021226001@tju.edu.cn (C.C.); yiyiyiyan610@163.com (Y.G.); myy-0706@163.com (Y.M.); 17888838150@163.com (Y.Z.); 13910096703@139.com (K.L.); 2 Beijing International Science and Technology Cooperation Base for Antiviral Drugs, Beijing Key Laboratory of Environmental and Viral Oncology, College of Chemistry and Life Science, Beijing University of Technology, Beijing 100124, China; zhailina8023@163.com; 3 Department of Cell Biology and Genetics, School of Basic Medical Sciences, Guangxi Medical University, 22 Shuangyong Road, Nanning 530021, China; 18877961233@163.com

**Keywords:** outer membrane vesicles, *Salmonella enterica* serovar Typhimurium, biocompatibility, adjuvant

## Abstract

Background: Outer Membrane Vesicles (OMVs), nanosized particles derived from Gram-negative bacteria, are promising vaccine carriers due to innate immunogenicity and self-adjuvant properties. Yet the systematic evaluations of OMV-associated toxicity remain limited. Methods: We developed a CRISPR/Cas9-engineered *Salmonella enterica* serovar Typhimurium *ΔmsbB* mutant (Mut4_STM) to produce detoxified OMVs (Mut4_OMVs) with enhanced yield. Subcutaneous immunization of BALB/c mice with Mut4_OMVs to evaluate safety, and the adjuvant efficacy was also determined by injecting Mut4_OMVs with *Yersinia pestis* F1Vmut or *Bacillus anthracis* PA_D4 antigens, mixing formulation, respectively. Results: Mut4_OMVs showed a 9-fold protein yield increase over wild-type OMVs. While all mice injected with wild-type OMVs died, 100% survival was observed in those receiving Mut4_OMVs. However, dose-dependent pathological alterations became evident in specific organs as the administration dose escalated, such as induced splenic extramedullary hematopoiesis and renal edema. Despite residual toxicity, 2–3 doses of 10 μg Mut4_OMVs elicited antigen-specific antibody titers comparable to aluminum adjuvant controls and superior T-cell immune responses. Conclusion: While Mut4_OMVs retain potent adjuvant activity, their residual toxicity necessitates further biocompatibility optimization for safe vaccine applications.

## 1. Introduction

The secretion of vesicles is an essential process that occurs in all cell types, facilitating and enabling cellular communication with the environment. Outer membrane vesicles (OMVs), which are derived from the cell envelope of Gram-negative bacteria, have been the subject of extensive research since their initial observation in an *Escherichia coli* strain in 1965. As vesicles derived from bacterial outer membranes, OMVs are composed of membrane proteins, membrane lipids, and cytoplasmic proteins while harboring cytoplasmic elements, like toxins, DNA, and RNA [1] in the lumen. Due to their pathogen membrane components and non-replicative nature, OMVs have attracted significant interest from researchers to explore their functions as antigens/drug carriers for tumors and diseases. Current research on OMVs focuses on the biogenesis mechanism [2], nano-vector of antigens for cancer therapies [3] or vaccine candidates [4,5].

Despite expressing heterogeneous antigens on their surface via fusion with certain membrane proteins, such as the autotransporter protein Hemoglobin protease (Hbp) [6,7,8], Cytolysin A (ClyA) [3,9,10,11], and Outer membrane protein A (OmpA) [12], OMVs can also serve as an adjuvant due to their cell-like structure [13,14]. Host immune responses serve as a barrier to prevent infections. Unlike pathogens, the nanoscale size of OMVs facilitates their uptake by antigen-presenting cells (APCs), thereby stimulating the innate immune system by activating the Toll-like receptors (TLRs) through various pathogen-associated molecular patterns (PAMPs), such as lipoproteins, lipopolysaccharides, and nucleic fragments [15]. Studies have shown that the OMVs derived from *Burkholderia pseudomallei* could function effectively as vaccine adjuvants and stimulate innate and antigen-specific adaptive immune responses when administered intramuscularly to mice. Furthermore, they have been shown to activate Dendritic Cells (DCs) both in vitro and in vivo [16,17].

Previous studies have demonstrated that the Tol-Pal system is essential for maintaining outer membrane integrity in Enterobacteriaceae [18], with targeted deletions of key genes, such as *tolR* [19], significantly enhancing OMV biogenesis. This genetic strategy has been successfully implemented in *Salmonella enterica* serovar Typhimurium (*S. typhimurium*, STM) [19]. OMVs-associated pathogen-associated molecular patterns (PAMPs) interact with various pattern recognition receptors (PRRs) on antigen-presenting cells (APCs). Specifically, Toll-like receptor 4 (TLR4) recognizes lipopolysaccharide (LPS), while flagellin activates both TLR5 and the NLR family CARD domain-containing protein 4 (NLRC4) [20,21]. Studies have shown that replacing the flagellin FliC of *Salmonella* with that of *Escherichia coli* (FliC_-K12_) can help bacteria evade Nod-like receptor CARD 4 (NLRC4) recognition, while NLRC4 suppresses the development of both effector and memory CD4 + T cell immunity. This finding suggests flagellin modification to reduce NLRC4 activation, which could have important implications for *Salmonella*-based vaccine development [22]. In addition, the *pagP* gene in STM encodes an acyltransferase responsible for lipid A palmitoylation, a critical determinant of LPS endotoxicity. Concurrently, MsbB mediates the biosynthesis of penta- and hexa-acylated lipid A species. Genetic ablation of both *msbB* and *pagP* significantly reduces the endotoxic potential of *Salmonella* LPS [23].

In this study, we investigated the adjuvant properties of outer membrane vesicles (OMVs) derived from *S*. *typhimurium*, a model organism extensively characterized in bacterial pathogenesis research. According to previous literature reports, we selected two protective antigen proteins, representing highly virulent pathogenic bacteria, as model antigens to evaluate this characteristic experimentally. PA_D4 (residues 596-735), corresponding to Domain IV of Bacillus anthracis protective antigen (UniProt P13423), has a molecular weight of approximately 17 kDa. Studies have demonstrated that the immunization of mice with PA_D4 as an antigen still provides effective protection against *Bacillus anthracis* challenge, demonstrating its potential as a vaccine antigen [24]. F1Vmut, a fusion protein comprising the fraction 1 and mutated V protein antigens of *Yersinia pestis*, has been widely used in the design and research of plague vaccines. It has been demonstrated to elicit potent immunoprotective efficacy [25]. Our results demonstrate that engineered bacterial OMVs can act as adjuvants, synergizing with target antigens to elicit robust humoral and cellular immune responses in experimental animals. Our work also provides experimental evidence for the development of a *Salmonella*-based bacterial OMVs vaccine platform.

## 2. Materials and Methods

### 2.1. Bacterial Strains and Growth

All bacterial strains used in the present study are listed in Table S1*. S. typhimurium* 1.1174 (wild type, WT-STM) was obtained from the China General Microbiological Culture Collection Center (CGMCC). The mutant strain, STM *ΔmsbB*, was stored in our laboratory. In this study, the quadruple mutant *S. typhimurium* 1.1174 Δ*msbB*Δ*tolR*Δ*pagP fliC*::*EcfliC^DH5α^* (Mut4_STM) was constructed using the CRISPR/Cas9 genome editing technology. Outer membrane vesicles (OMVs) were derived from Mut4_STM. *E. coli* BL21 (DE3) was utilized for antigen protein expression. All bacterial strains were cultured in lysogeny broth (LB) at 37 °C (LB composition: 1% (*w*/*v*) tryptone, 1% (*w*/*v*) NaCl, and 0.5% (*w*/*v*) yeast extract).

### 2.2. Construction of Mut4_STM

All plasmids used in the present study are listed in Table S1. Based on the STM Δ*msbB* strain, we sequentially deleted the genes *tolR* and *pagP* from its genome. In this process, the plasmids pCas9 and pTargetA were utilized, which confer kanamycin resistance (Kan^R^) and ampicillin resistance (Amp^R^), respectively. The pTargetA plasmid was derived from pTargetF (Addgene #62226) by replacing the original spectinomycin resistance gene with an ampicillin resistance gene, and it was subsequently stored in our laboratory. To construct the recombinant strain for OMVs production with high yield and low toxicity, *tolR* and *pagP* were selected as knockout targets according to the published work [18,23,26]. These genes were deleted or replaced following the protocol established by Jiang Y [27]. Briefly, using the *tolR* gene as an example, it was replaced by a DNA fragment, designated as *tolR_Rp*, which consists of its upstream and downstream regions. Initially, the *tolR_Rp* DNA fragment was synthesized and inserted into a plasmid by Gencefe (Beijing, China), followed by PCR amplification using the primer pairs *tolR_Rp_Fw/Rv* (Table 1). The guide RNA (gRNA) targeting *tolR* (*gtolR*) was inserted into pTargetA by PCR using the primer pairs *tolR_n20_Fw/sg_R*, which included a BsaI restriction site. The linearized plasmid was then circularized using the Golden Gate Assembly Kit BsaI-HFv2 (New England Biolabs, Ipswich, MA, USA) and cloned into DH5α competent cells. Subsequently, pCas9, pTargetA_gtolR, and the *tolR_Rp* fragment were electroporated sequentially into STM *ΔmsbB* competent cells in two steps (pCas9 first, followed by pTargetA and the *Rp* fragment, requiring two separate electroporation steps). The successful deletion of the *tolR* gene was confirmed through PCR, nucleic acid electrophoresis, and sequencing. The editing of the *pagP* and *fliC* genes was carried out using the same methodology. At the same time, the *fliC* gene was replaced by orthologs of *fliC* from *E. coli* DH5α, being designated as *EcfliC^DH5α^*.

### 2.3. Isolation of Outer Membrane Vesicles (OMVs)

The Mut4_STM strain was cultured overnight in LB broth at 37 °C. On the subsequent day, 1.5 mL of the overnight culture was transferred into 15 mL of fresh LB medium and incubated until the optical density at 600 nm (OD_600_) reached 1.0. This freshly cultured Mut4_STM was then diluted at a ratio of 1:100 (*v*/*v*) into 1 L of fresh LB medium and incubated at 37 °C for an additional 5 h. Post-incubation, the culture underwent centrifugation at 8000× *g* for 10 min to collect the supernatant, which was subsequently filtered through a 0.45 μm Mixed Cellulose Ester membrane filter (NEST, Wuxi, China). And the filtered medium was further concentrated using a 100 kDa ultrafiltration unit (Sartorius AG, Göttingen, Germany) and transferred to ultracentrifuge tubes. The samples were ultracentrifuged at 150,000× *g* for 4 h using a Beckman ultracentrifuge (Beckman, Germany). After ultracentrifugation, the supernatant was carefully removed, and the pellet at the bottom of the tubes was resuspended in 1 mL of saline solution and filtered through a 0.22 μm membrane (Pall Corporation, Port Washington, NY, USA). The resuspended OMVs were stored at −80 °C for further use. OMVs derived from WT-STM (WT_OMV) were isolated following the same protocol.

### 2.4. Characterization of WT_OMVs, Mut4_STM and Mut4_OMVs

The Mut4_STM strain and both types of OMVs (including WT_OMV and Mut4_OMV) were fixed and stained prior to electron microscopy imaging. After separating the Mut4_STM pellets and culture supernatant, the pellets were collected and fixed in 4% glutaraldehyde (Solarbio, Beijing, China) for 24 h, ensuring minimal disturbance to the bacterial pellets. Then, the well-fixed bacterial samples were prepared in ultrathin sections for observation experiments. For the OMVs, the pellets were diluted properly, for instance, ten-fold, with saline and mixed in a 1:1 (*v*/*v*) ratio with 4% glutaraldehyde for 30 min. The fixed OMV samples were then transferred onto carbon-coated copper grids (EMCN, Nanjing, China) and allowed to settle for 10 min. Excess liquid was carefully removed using filter paper. The grids were then stained with 1% phosphotungstic acid for 1 min, after which any residual dye was gently blotted away with filter paper and allowed to dry; then, the OMV sample was observed using a transmission electron microscope HT7800 (Hitachi, Tokyo, Japan).

### 2.5. Protein Quantification and Particle Size Analysis

The yield of outer membrane vesicles (OMVs) was quantified using a BCA assay kit (Tiangen, Beijing, China), following the manufacturer’s instructions. Proteins were stained after SDS-PAGE using eStain (GenScript, Nanjing, China) and transferred to PVDF membranes by eBlot (Genescript, China), followed by blocking with 5% non-fat milk in PBST. Membranes were incubated with Rabbit Polyclonal antibodies to flagellin (Abcam, Cambridge, UK) (1:5000 dilution) at room temperature (RT) for 1 h, then with HRP-conjugated secondary antibody (1:5000) for 1 h at RT. Signals were developed using SuperSignal West Pico Chemiluminescent Substrate (Thermo Fisher Scientific, Waltham, MA, USA) and captured with a ChemiDoc MP Imaging System (Bio-Rad Laboratories, CA, USA). Additionally, the size of the OMVs was determined using a Zetasizer Pro (Malvern Panalytical, Malvern, UK). OMV pellets were diluted tenfold with saline to a volume of approximately 1.2 mL before being added to the cuvettes for analysis with the Zetasizer.

### 2.6. Biocompatibility Analysis

Female BALB/c mice, aged 6–8 weeks, were procured from Charles River (Beijing, China) for the study. All animal experiments were conducted with the permission of the Animal Care and Use Committee at the Academy of Military Medical Sciences under Ethical Approval No. IACUC-DWZX-2024-554. To evaluate the safety profile of OMVs derived from a mutant strain, a comparative analysis was performed between Mut4_OMV and wild-type OMVs (WT_OMVs). Mice were divided into four groups (*n* = 5 per group) and administered OMVs subcutaneously at varying doses with a 14-day interval: WT_OMV (10 μg) for one dose, Mut4_OMV (10 μg) for three doses, Mut4_OMVs (30 μg) for two doses, and Mut4_OMVs (50 μg) for two doses. The clinical status of the mice was recorded, including changes in weight, temperature, behavior, and appearance.

### 2.7. Evaluation of Hepatic and Renal Function

Blood samples were collected from mice 35 days following the final dose administration. The collection was performed from the retro-orbital plexus under isoflurane anesthesia using heparinized capillary tubes. Subsequently, the mice were euthanized via intraperitoneal injection of sodium pentobarbital at a dosage of 150 mg/kg body weight. The collected blood samples were centrifuged at 3000× *g* at room temperature for 15 min to separate the sera. All serum samples were sent to Servicebio Technology (Wuhan, China) for analysis. The concentrations of Lactate Dehydrogenase (LDH), alanine aminotransferase (ALT), aspartate aminotransferase (AST), alkaline phosphatase (ALP), and blood urea (UREA) were quantified.

### 2.8. Organ Harvesting and H&E Staining

Livers, spleens, and kidneys of mice from biocompatibility analysis groups (Section 2.6) were collected after being euthanized. All organ samples were fixed in 4% paraformaldehyde for 24 h. Then, the sections and hematoxylin and eosin (H&E) staining were performed by Servicebio Technology (Wuhan, China).

### 2.9. Expression and Purification of Antigen Protein

The coding sequence of the *B. anthracis* protective antigen domain 4(PA_D4) was synthesized by Gencefe Biotech (Wuxi, China) and subsequently cloned into the expression vector pET28a (+) using the NcoI and XhoI restriction sites, resulting in the plasmid pET28a (+)-PA_D4. The constructed plasmid was then transformed into *E. coli* BL21 (DE3) for protein expression. The expression strain was cultured in LB medium at 37 °C until the OD_600_ reached 0.6–0.8. Protein expression was induced by the addition of 1 mM isopropyl β-D-1-thiogalactopyranoside (IPTG), followed by incubation overnight at 30 °C. The bacterial cells were harvested the following day through centrifugation at 8000× *g* for 15 min and stored at −20 °C for further processing. The PA_D4 protein was purified under native conditions using a HisTrap HP column (Cytiva, Marlborough, MA, USA). F1Vmut Protein was kindly provided by Dr. Ting Fang from the Beijing Institute of Biotechnology.

### 2.10. Mice Vaccination

Mice were divided into 6 groups, 10 per group, and immunized subcutaneously with two types of formulation in a volume of 100 μL: OMVs + antigen protein, adjuvant + antigen protein, and antigen-only. Containing 10 μg Mut4_OMV + 1 μg F1Vmut, or 100 μg Alhydrogel (Croda, Snaith, UK) + 1 μg F1Vmut, 10 μg Mut4_OMV + 20 μg PA_D4, and 100 μg Alhydrogel + 20 μg PA_D4, 1 μg F1Vmut, and 20 μg PA_D4. The immunization schedule consisted of two doses for F1Vmut and three doses for PA_D4, with two-week intervals between each dose.

### 2.11. Antigen-Specific Enzyme-Linked Immunosorbent Assay (ELISA)

Antigen-specific antibodies present in sera were quantified utilizing an enzyme-linked immunosorbent assay (ELISA). Costar^®^ assay plates (Corning Incorporated, Corning, NY, USA) were coated with 1 μg/mL of purified PA_D4 or F1Vmut in a carbonate buffer (pH 9.6) and incubated overnight at 4 °C. The plates were then blocked with 1% BSA blocking buffer (100 μL/well; buffer composition: PBS, 0.05% (*v*/*v*) Tween-20, and 1% (*w*/*v*) BSA) for 1 h at 37 °C. Samples were added in serial 2-fold dilutions (100 μL/well) and incubated for 1 h at 37 °C. This was followed by a 1-h incubation with HRP-conjugated anti-mouse immunoglobulin G (IgG), IgG1, or IgG2a antibodies (Abcam, Cambridge, UK, dilution 1:50,000) at 37 °C. After washing the plates three times with PBST, the reaction was developed using a tetramethylbenzidine (TMB) solution (Solarbio, Beijing, China) for 6 min. The reaction was stopped by adding 2M H_2_SO_4_. The optical density (OD) was measured using an iMax3 microplate reader at wavelengths of 630 nm and 450 nm.

### 2.12. Splenic Lymphocyte Proliferation and Cytokine Quantification

To assess the splenic lymphocyte proliferation and cytokine production of antigen-specific, spleens were harvested twenty-eight days after the last immunization. The splenic tissues were processed into single-cell suspensions by passing through a 70 μm cell strainer. Lymphocytes were isolated using a splenic lymphocyte isolation kit ( Solarbio, Beijing, China). Lymphocytes (~4 × 10^6^ cells) from mice administrated antigen protein (F1Vmut and/or PA_D4) + Mut4_OMV were incubated for 72 h with or without 1 μg F1Vmut and 5 μg PA_D4 in RPMI 1640 medium. During the final 4 h of incubation, CCK-8 solution was added, and the OD was measured at 450 nm using a SpectraMax I3X (Molecular Devices, San Jose, CA, USA). Subsequently, cell culture supernatants were collected for cytokine analysis, employing the Ella Automated ELISA (Bio-Techne Corporation, Minneapolis, MN, USA). Cytokines such as Interferon gamma (IFN-γ), interleukin-6 (IL-6), IL-2, IL-4, and tumor necrosis factor-alpha (TNF-α) were quantified.

### 2.13. Statistical Analysis

Data are shown as mean values plus the standard error of the mean (SEM). Statistical significance of differences between OMV + PA_D4/OMV + F1Vmut and OMV-Ctrl vaccinated groups was determined using the analysis of variance (ANOVA) and Sidak’s multiple comparisons test in GraphPad Prism version 8.0. Levels of statistical significance are denoted by asterisks as * *p* < 0.05, ** *p* < 0.01, *** *p* < 0.001, and **** *p* < 0.0001.

## 3. Results

### 3.1. Construction of Mut4_STM Strain

**Figure 1:**
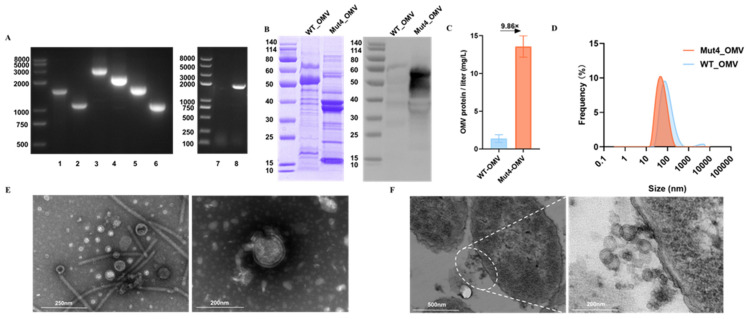
Generation of WT_OMVs and Mut4_OMVs and their comparison. (**A**) The PCR products of mutated genes (*msbB*, *tolR*, *pagP*, and *EcfliC^DH5α^*) were analyzed by agarose gel electrophoresis. 1: Δ*tolR*-negative (1544 bp); 2: Δ*tolR*(1115 bp); 3: Δ*msbB*-negative (~3000 bp); 4: Δ*msbB* (2000 bp); 5: Δ*pagP*-negative (1726 bp); 6: Δ*pagP* (1153 bp); 7: *EcfliC*^DH5α^-negative (no bands); 8: *fliC*::*EcfliC^DH5α^* 1659 bp); (**B**) SDS-PAGE and western blot analysis. (20 μg protein in each lane). (**C**) The yield of Mut4_OMV and WT_OMV was quantified using the BCA method. (**D**) The particle size of Mut4/WT OMV was measured using a dynamic light scattering system (DLS). (**E**) WT_OMVs showed round-shaped vesicles surrounded by flagella (left). And Mut4_OMVs vesicles have bilayer membranes (right). (**F**) Mut4_STM stain produces numerous OMVs.

To improve OMVs’ yield, reduce endotoxicity, and optimize endogenous flagellin utilization, we engineered a derivative of the STM Δ*msbB* strain. The *tol-pal* system has been extensively studied in various Gram-negative bacteria, where genes associated with this system influence the integrity of the outer membrane, thereby promoting the production of OMVs. Additionally, previous studies have identified the *msbB* and *pagP* genes as contributors to the biosynthesis of fully penta-acylated lipid A in *S. typhimurium*. The lipid A component also plays a significant role in modulating reactogenicity and immunogenicity. To develop an OMV with reduced virulence and increased blebbing compared to the wild-type OMV, we engineered the STMΔ*msbB* strain by deleting the chromosomal genes *tolR* and *pagP*. The successful deletion of these three genes was confirmed through PCR (Figure 1A) and sequencing, utilizing primers dtolR_Rp_Fw/Rv, dpagP_Rp_Fw/Rv, and rfliC_Rp_Fw/Rv. Consequently, we generated triple knockout and *fliC*::*EcfliC^DH5α^* mutants of STM.

### 3.2. Isolation and Characterization of OMVs from Mut4_STM

Following the collection of outer membrane vesicles (OMVs), the protein profile was analyzed using Coomassie blue staining. And western blotting with anti-flagellin antibodies verified the successful replacement of the fliC gene (Figure 1B). The major protein(s) in WT_OMVs migrated at ~50 kDa, while the Mut4_OMVs were about 40 kDa [28]. The yield of OMVs increased more than ninefold (Figure 1C), and the size distribution of Mut4_OMV was assessed using dynamic light scattering analysis, revealing a size range of 30 to 100 nm (Figure 1D). Transmission Electron Microscopy (TEM) was employed to verify the morphology of Mut4_STM and the OMVs. TEM images of Mut4_STM demonstrated vesicles encircling the bacterial cells, characterized by a distinctly visible double-layer structure. This observation enabled the identification of OMVs from the culture supernatant pellets. Similarly, Mut4_OMV exhibited a double-layer membrane structure under electron microscopy, with the majority of OMV particles appearing spherical and resembling the vesicles adjacent to the bacterial cells. Subsequently, we evaluated the particle size distributions and surface charges of Mut4_OMV to further elucidate their properties (Figure 1E,F).

### 3.3. The Safety of Mut4_OMV

**Figure 2:**
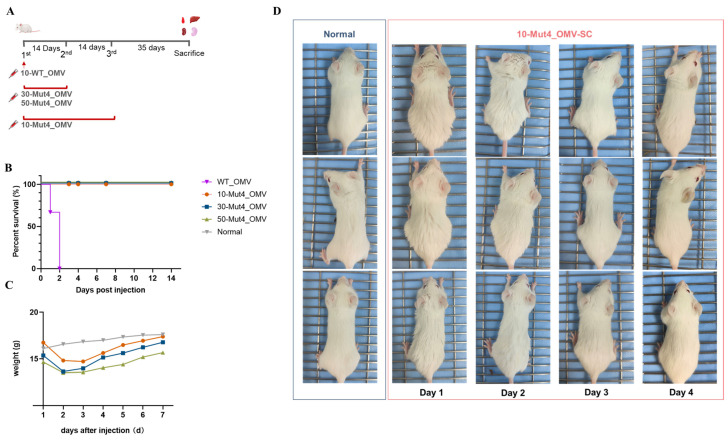
Safety evaluation of Mut4_OMVs. Survival rates were monitored in mice following intraperitoneal injection of Mut4_OMVs versus WT_OMVs (**A**), with body weight changes (**B**) and clinical status (**C**) of normal and 10-Mut4_OMVs-SC mice (*n* = 5) recorded during the first post-injection week. Notably, injected mice developed piloerection and weight loss associated with reduced appetite. The status of the mice in the 10 μg dose injection group was shown visually (**D**).

To assess the safety profile of Mut4_OMVs, a comparative analysis was conducted between Mut4_OMV and WT_OMV (Figure 2A). Mice were allocated into four groups (*n* = 5 per group) and administered WT_OMVs and Mut4_OMVs in different doses subcutaneously. The WT_OMVs resulted in severe symptoms, leading to the death of all mice within two days. In contrast, all mice receiving Mut4_OMVs survived (Figure 2B). Although Mut4_OMVs did not cause mortality, mice exhibited adverse symptoms, which were dose-dependent, including piloerection and signs of lethargy or sluggishness, characterized by a marked reduction in activity during the first week post-administration (Figure 2D). This initially resulted in weight loss among the mice (Figure 2C). Symptoms in the 10 μg Mut4_OMVs group alleviated within two days, whereas symptoms persisted longer in the 30 μg and 50 μg groups, with notable eye discharge observed in three out of five mice (Figure S1C), a symptom that was not present in the 10 μg group (Figure S1A).

**Figure 3:**
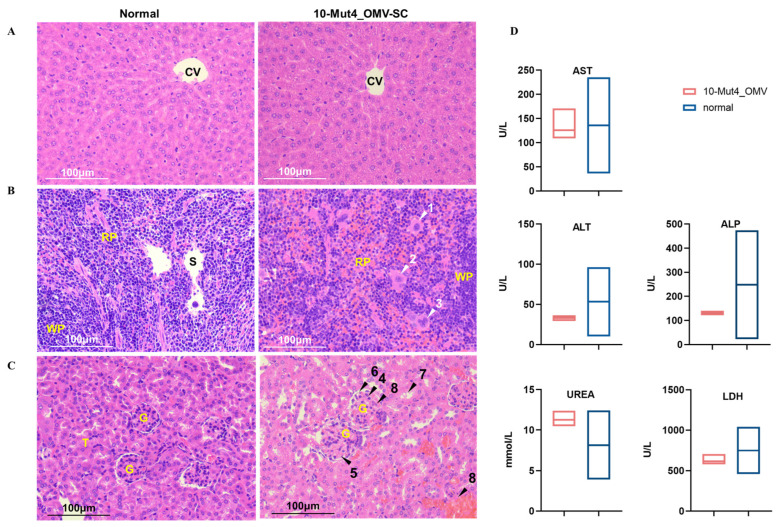
Histopathological analysis. H&E-stained liver sections from 10-Mut4_OMV mice revealed no significant pathological changes (**A**). Megakaryocytes (M) were identified within the splenic red pulp (RP) (**B**). Normal group showing the normal glomerulus (G), and tubules (T); and OMV group showing cytoplasmic swelling and vacuolar changes affecting both glomeruli and surrounding structures (black arrow) (**C**). And levels of LDH, ALT, AST, ALP, and UREA of 10-Mut4_OMV mice (*n* = 5) (**D**). CV: central vein; S: sinusoid; WP: white pulp; RP: Red pulp; G: glomerulus; T: tubules.

Upon postmortem gross examination, the livers and spleens of mice administered with WT_OMVs and Mut4_OMVs exhibited hepatosplenomegaly, with more pronounced enlargement observed in the WT, Mut4_30, and Mut4_50 groups compared to healthy controls. The group administered 10 μg Mut4_OMVs showed the mildest symptom (Figure S2). Histopathological examinations were conducted on major organs, including sections of the liver, spleen, and kidneys, to further evaluate the effects. Liver sections from the 10-Mut4_OMVs group showed no significant pathological alterations (Figure 3A). Inflammatory cell infiltrates around the central vein (CV) were observed in livers from 30 and 50-Mut4_OMVs group mice (Figure S2). In contrast, the spleens exhibited an increased, pink-stained area in the red pulp, indicative of expanded erythrocyte distribution. Megakaryocytes were identified in the red pulp (indicated by arrows 1, 2, 3 in Figure 3B and Figure S2). The increased erythrocyte count in the spleen suggests splenic congestion, characterized by localized vascular dilation and erythrocyte accumulation, which is consistent with the observed enlargement of spleens in the 10-Mut4_OMVs group during dissection, confirming the presence of splenic congestion. As illustrated in Figure 3C, the 10-Mut4_OMVs group exhibited lighter overall kidney staining compared to the normal group (Figure 3C left panel). The glomeruli demonstrated increased volume (indicated by arrow 4), the presence of cytoplasmic vacuoles of varying sizes (arrow 5), and significantly widened spaces between the glomeruli and the capsule walls (arrow 6). Renal tubular epithelial cells showed signs of swelling, pale cytoplasmic staining, and partial cellular blurring (arrow 7). Additionally, serum levels of lactate dehydrogenase (LDH), alanine aminotransferase (ALT), aspartate aminotransferase (AST), alkaline phosphatase (ALP), and blood urea (UREA) were measured, revealing that liver and kidney function in the 10-Mut4_OMV-vaccinated mice remained within normal ranges (Figure 3D). These findings suggest that a 10 μg dose of Mut4_OMVs demonstrates safety, with animals exhibiting potential recovery over time.

### 3.4. The Mut4_OMVs Show the Adjuvant Properties

**Figure 4:**
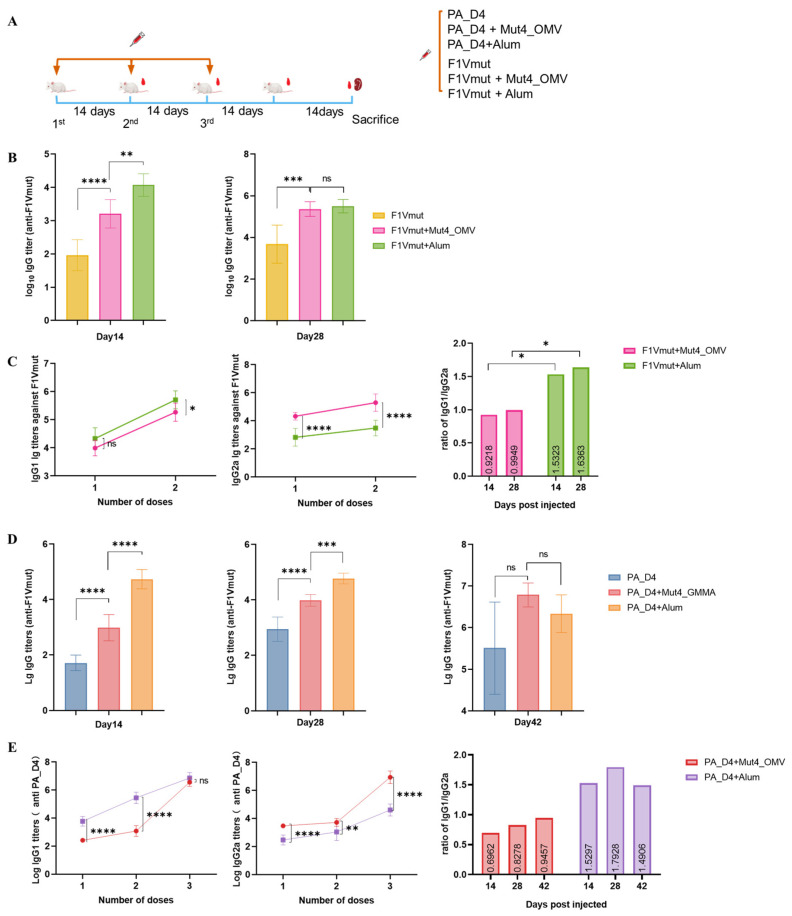
Assessment of humoral immune responses. Mice were subcutaneously administered 10 μg Mut4_OMVs or 100 μg Alum + 1 μg F1Vmut, 10 μg Mut4_OMVs or 100 μg Alum + 20 μg PA_D4, 1 μg F1Vmut alone, and 20 μg PA_D4 alone every 2 weeks. Serum was isolated every 2 weeks (**A**), and F1Vmut and/or PA_D4-specific IgG (**B**,**D**), the ratio of IgG1/IgG2a titer (**C**,**E**) were measured by ELISA. Differences between groups were tested using one-way ANOVA. ns *p* > 0.05. * *p* <0.05, ** *p* <0.01, *** *p* < 0.001 and **** *p* < 0.0001.

To investigate the capacity of OMVs-based vaccines to induce humoral responses, female BALB/c mice were immunized with Mut4_OMVs combined with F1Vmut or PA_D4 protein. Control groups of mice received immunizations as follows: Alum with F1Vmut, Alum with PA_D4, F1Vmut alone, and PA_D4 alone. Blood samples were collected on days 14, 28, and 42. The specific antibody titers against F1Vmut and PA_D4 in serum were measured using indirect ELISA. To measure the level of humoral immunity, we examined antigen-specific IgG, IgG1, and IgG2a levels. As shown in Figure 4B,D.

Following the initial injection, all experimental groups exhibited low levels of anti-F1Vmut or anti-PA_D4 antibody responses, with titers below 1:1500 (Figure 4B,E, day 14). Notably, the group receiving adjuvant treatment demonstrated significantly higher anti-F1Vmut responses, whereas no such increase was observed for PA_D4. The anti-F1Vmut antibody responses were markedly enhanced following the second booster (Figure 4B, day 28), comparable to the Alum group, indicating that the booster was essential for achieving an adequate immune response. Furthermore, after the third booster, the IgG titers in mice administered with Mut4_OMVs + PA_D4 were substantially higher than those in mice administered with Alum + PA_D4 (Figure 4E, day 42). Compared to the group that received only F1Vmut, the Mut4_OMVs-vaccinated mice exhibited significantly elevated levels of F1Vmut (or PA_D4)-specific IgG titers.

To preliminarily evaluate the type of immune response elicited by Mut4_OMVs, we measured the titers of IgG subtypes, specifically IgG1 and IgG2a. Notably, compared to the Alum-adjuvanted group, Mut4_OMVs demonstrated a preferential induction of IgG2a relative to IgG1. These results indicate that Mut4_OMVs effectively stimulate a humoral immune response against both antigens, achieving comparable efficacy to the aluminum adjuvant. The IgG1/IgG2a ratio analysis (Figure 4C,E) revealed distinct patterns: 0.9948 in the F1Vmut + Mut4_OMVs group, and 0.8379 (second dose) versus 0.9457 (third dose) in the PA_D4 + Mut4_OMVs group. This elevated production of IgG2a suggests that Mut4_OMVs may enhance Th1-mediated immune differentiation. However, comprehensive characterization of the Th1/Th2 bias will require additional investigations, such as cytokine profiling combined with T cell subset analysis.

### 3.5. Analysis of the Immune Response Induced by Mut4_OMVs

**Figure 5:**
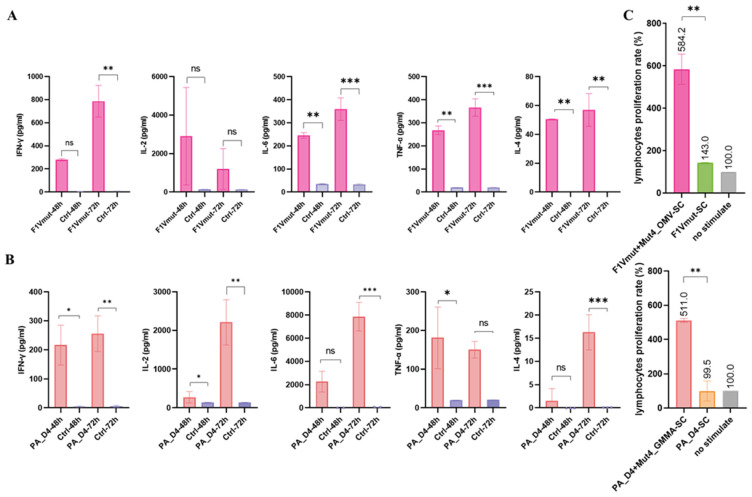
Cytokine production by splenic lymphocytes following antigen stimulation was measured, including Levels of IFN-γ, IL-2, IL-6, TNF-α, and IL-4, to evaluate the immune responses. Spleens of mice from 4 groups: Group F1Vmut includes mice immunized with F1Vmut + Mut4_OMVs and F1Vmut-alone (**A**). Group PA includes mice immunized with PA_D4 + OMVs and PA_D4-alone (**B**). Splenocytes were stimulated in vitro with F1Vmut or PA_D4. The lymphocyte proliferation rates were also measured (**C**). Differences between groups were tested using one-way ANOVA. ns *p* > 0.05. * *p* < 0.05, ** *p* < 0.01, and *** *p* < 0.001.

To evaluate the immunogenicity of the antigen protein formulations (F1Vmut or PA_D4) in combination with Mut4_OMVs, mice were immunized with either the antigen alone or the antigen in conjunction with Mut4_OMVs. Following the final immunizations over 35 days, the mice were euthanized to collect spleens for subsequent analyses. We assessed the rate of lymphocyte proliferation and secretion of cytokines IFN-γ, IL-2, TNF-α, IL-6, and IL-4 by splenic lymphocytes. As depicted in Figure 5A,C, cytokine expression was elevated in both OMVs-vaccinated groups compared to the control group (unstimulated cells). Notably, the expression levels of IFN-γ, IL-2, TNF-α, and IL-6 were significantly increased, with IL-6 showing a pronounced elevation in the PA_D4 group, and IL-2 in both groups. The marked increase in IL-2 and IFN-γ suggests an upregulation of cytokines predominantly produced by the Th1 cell subset, which in turn naturally suppresses the secretion of IL-4. Furthermore, it is well-established that activated T cells, B cells, and natural killer (NK) cells are capable of secreting IL-6 and TNF-α, which may account for the observed elevation in these cytokines. Furthermore, we assessed the effects of the combined vaccine formulations on the proliferation of splenic lymphocytes in mice (Figure 5B,C). Our results revealed that antigen-stimulated cells showed significantly enhanced proliferation compared to unstimulated controls. These findings suggest that subcutaneous co-administration of Mut4_OMVs with the antigen protein induces strong immune responses, including both cellular and humoral immunity.

## 4. Discussion

In this study, the feasibility of Mut4_STM OMVs from the mutated STM used as a vaccine adjuvant was investigated. We assessed the safety profile of Mut4_OMVs and demonstrated its humoral immunogenicity when co-administered with antigens in murine models, as evidenced by the detection of antigen-specific antibody titers and cytokine secretion following splenocyte stimulation. By evaluating various dosages of Mut4_OMVs injections in mice, alongside physiological observations, histopathological examinations of major organs, and biochemical analyses of hepatic and renal functions, we identified an optimal immunization a dosage of 10 μg per mouse was optimal for immunization. Subsequently, Mut4_OMVs were co-administered subcutaneously with two antigenic proteins, F1Vmut and PA_D4, in mice. The results indicated that Mut4_OMVs exhibited adjuvant activity by effectively inducing antigen-specific antibodies, with titers comparable to those elicited by conventional aluminum adjuvants. Analysis of IgG subtypes suggested that Mut4_OMVs may preferentially promote T-cell differentiation into Th1 cells. Upon antigenic stimulation of splenic lymphocytes, cytokine profiling of the culture supernatants revealed a predominant production of Th1-associated cytokines, such as IFN-γ and IL-2. These results further substantiate the preliminary conclusions drawn from cellular subtype analysis, confirming that Mut4_OMVs facilitates Th1-biased T-cell differentiation.

The genetic modification (Δ*pagP*Δ*msbB*) in *S. typhimurium*, which promotes the synthesis of less toxic penta-acylated lipid A by turning off lipid A acyltransferases [23], significantly improved survival rates in mice (Figure 2B). However, Mut4_OMVs also triggered adverse effects, including piloerection (Figure 2C), excessive ocular secretions, and reduced appetite (Figure S2). In the cohort administered a 10 μg dose, these symptoms persisted for three days before subsiding. Notably, histopathological analysis of major organs via H&E staining on day 35 post-immunization revealed unresolved tissue damage, such as cellular edema, erythrocyte accumulation in red pulp, and extramedullary hematopoiesis (Figure 3A–C). The presence of megakaryocytes in the spleen (indicated by arrows in Figure 3B), typically restricted to bone marrow for platelet production, might support compensatory extramedullary hematopoiesis—a response linked to ischemia or hematopoietic dysfunction. Although serum lactate dehydrogenase (LDH) levels remained normal, these pathological findings imply residual toxicity in the Mut4_OMV formulation, underscoring unresolved safety concerns. Surprisingly, previous studies using Mut4_OMVs neither implemented additional detoxification strategies nor reported comparable adverse reactions post-vaccination. And our finding underscores the necessity of incorporating virulence assessment protocols early in OMV-based vaccine development in the future [29,30,31]. Although addressing toxicity mitigation is beyond the scope of this study, further research is essential to enhance the safety of OMVs for therapeutic applications. Potential strategies could involve modifying culture media conditions to reduce toxicity or decreasing the administered dose.

OMVs are capable of triggering both innate and adaptive immune responses through various routes, such as intranasal and subcutaneous delivery. Huynh et al. determined that murine dendritic cells (DCs) can efficiently recognize and internalize OMVs, leading to DC activation and maturation [32]. We conducted an investigation into the adjuvant properties of Mut4_OMVs. Mice were subcutaneously immunized with Mut4_OMVs in combination with two antigenic proteins, F1Vmut and PA_D4. The antigen-specific antibody titers in serum indicated that serum antibody titers following two immunizations with F1Vmut + Mut4_OMVs were comparable to those achieved with the conventional aluminum adjuvant. And PA_D4 required three immunizations to reach a similar level of efficacy as the aluminum adjuvant. The IgG1/IgG2a ratio of Mut4_OMV-immunized groups suggested an immune response extending beyond humoral immunity (Figure 4C,E; IgG1/IgG2a ratio < 1). Implied that the immune system of mice administered with Mut4_OMVs tended to differentiate into T-helper 1 (Th1) types of cells. In comparison to non-immunized control groups, mice immunized with Mut4_OMVs exhibited elevated cytokine levels in IFN-γ, IL-2 (Figure 5A,B), and the splenic lymphocytes were significantly (Figure 5C). The observed increase in IFN-γ levels is likely responsible for inducing monocytes/macrophages to produce IL-6, IL-8, IL-2, and TNF-α [33,34]. Given that circulating monocytes predominantly reside in the bloodstream and splenic lymphocytes contain a limited number of tissue-resident macrophages, it is plausible that other IL-2-activated cells, such as NK/NKT cells, contribute to the production of IL-6 and TNF-α [33,34,35]. Activated natural killer (NK) cells have the capability to secrete IFN-γ and IL-4, thereby modulating immune polarization. IFN-γ plays a critical role in inhibiting T-helper 2 (Th2)-mediated IL-4 production and counteracting the effects of IL-4 on B cells. This results in the suppression of immunoglobulin E (IgE) production while promoting IgG synthesis. Our findings of elevated IgG antibody titers and relatively low IL-4 levels, despite their increase compared to control groups, may be attributed to the suppressive effects of IFN-γ. Nonetheless, the precise cellular sources of these cytokines remain speculative, based on current immunological understanding. To confirm immune cell activation, the following methodologies are proposed: (a) Isolate stimulated lymphocytes using flow cytometry, focusing on CD3+CD4+ T cells, and assess IL-2 receptor expression along with Th1 markers (CCR5+CXCR3+) [36] to verify the predominance of Th1 cells as the primary source of IL-2 and IFN-γ. (b) detected CD23 and MHC class II in B cells [37].

## 5. Conclusions

In conclusion, this study presents initial evidence suggesting that Mut4_OMV exhibits both humoral immunostimulatory capacity and cellular immune-activating potential. While the immunostimulatory properties of Mut4_OMV have been empirically established, its immunization efficacy requires comprehensive experimental validation. Furthermore, establishing an optimal dosing regimen necessitates further investigation to reconcile immunopotency with biosafety parameters.

## Figures and Tables

**Table 1:** Primer sequences used in this study.

Primers	Sequence
*tolR_Rp_Fw*	GTACCAGGAAAGCCAGGGGAG
*tolR_Rp_Rv*	TTTCTTCGCGTCCGCCGCCG
*pagP_Rp_Fw*	GCTGATAATAAGCAGAGCCAGACCG
*pagP_Rp_Rv*	CGTTTGCCATGACGGCGCTGG
*fliC_Rp_Fw*	GTTTTTTCGCTGAGTGCCAT
*fliC_Rp_Rv*	AGATCTTCAGTGGTGCTGGA
*tolR_n20_Fw*	CCAGGTCTCAGTCCTAGGTATAATACTAGTCAAGTCGACTTCCACGCTCTGTTTTAGAGCTAGAA
*pagP_n20_Fw*	CCAGGTCTCAGTCCTAGGTATAATACTAGTGCTGGGACGACAAAGGCAAC GTTTTAGAGCTAGAA
*fliC_n20_Fw*	CCAGGTCTCAGTCCTAGGTATAATACTAGTTCATCTGCAGTGTATTTCGT GTTTTAGAGCTAGAA
*sg_R*	CCAGGTCTCAGGACTGAGCTAGCTGTCAAGGATCCAGCATATGCGG
*dtolR_Rp_Fw*	GCGAACGCGTATTCTGAACGCC
*dtolR_Rp_Rv*	CGCTTTCGCCGCCTCGGCC
*dpagP_Rp_Fw*	TGATGTATTCAACAATATCTGTTGCGG
*dpagP_Rp_Rv*	TGCTGCAAGAGGGGCGCTTTG
*rfliC_Rp_Fw1*	GGCATACACCTGTTCCAGTT
*rfliC_Rp_Rv1*	CCAGTAACTGCTTTTGGTGC
*rfliC_Rp_Fw2*	CTAACAGCACCAAGGTTGGC
*rfliC_Rp_Rv2*	AGATCTTCAGTGGTGCTGGA

Note: letters in red are the n20 sequences, and letters in blue are the sequences of the pTargetA plasmid.

## Data Availability

The dataset is available from the authors.

## Supplementary Materials

The following supporting information can be downloaded at https://www.mdpi.com/article/10.3390/vaccines13060552/s1: Figure S1: Systemic responses in mice following injection with 30 μg or 50 μg Mut4_OMVs. Figure S2: Histopathology of the liver, spleen, and kidney from group 30- and 50-Mut4_OMV mice. Table S1: Overview of the bacterial strains and plasmids used in this study.
