# Supplementary material for: Engineered Outer Membrane Vesicles for Antigen Delivery: Exploratory Study on Adjuvant Activity and Systemic Reactogenicity
Source: Vaccines (Basel). 2025 May 22;13(6):552. doi: 10.3390/vaccines13060552 (PMC12197509; doi:10.3390/vaccines13060552)
Supplement: Supplementary file 1 [file vaccines-13-00552-s001.zip › vaccines-3579073-supplementary.pdf]

vaccines

MDPI

Table S1. overview of the bacterial strains and plasmids used in this study.

Plasmid or Strain	Description	Source of Reference
plasmids		
pTargetA	derived from plasmid pTargetF(Addgene #62226)), ampicillin resistance gene was used instead of spectinomycin resistance gene	lab stock.
pCas9	contains Cas9 enzyme, arabinose promoter, kanamycin resistance	Addgene #62225
pET28a(+)	for expression of PA_D4, T7 promoter, kanamycin resistance	lab stock
strains		
S. Typhimurium 1.1174	Wild-type strain	CGMCC
S. Typhimurium Δ*msbB*	*S. Typhimurium* Δ*msbB*	lab stock
Mut4_STM	*Salmonella Typhimurium 1.1174* Δ*msbB* Δ*tolR* Δ*pagP fliC*::*EcfliC*$^{DH5\alpha}$	this study

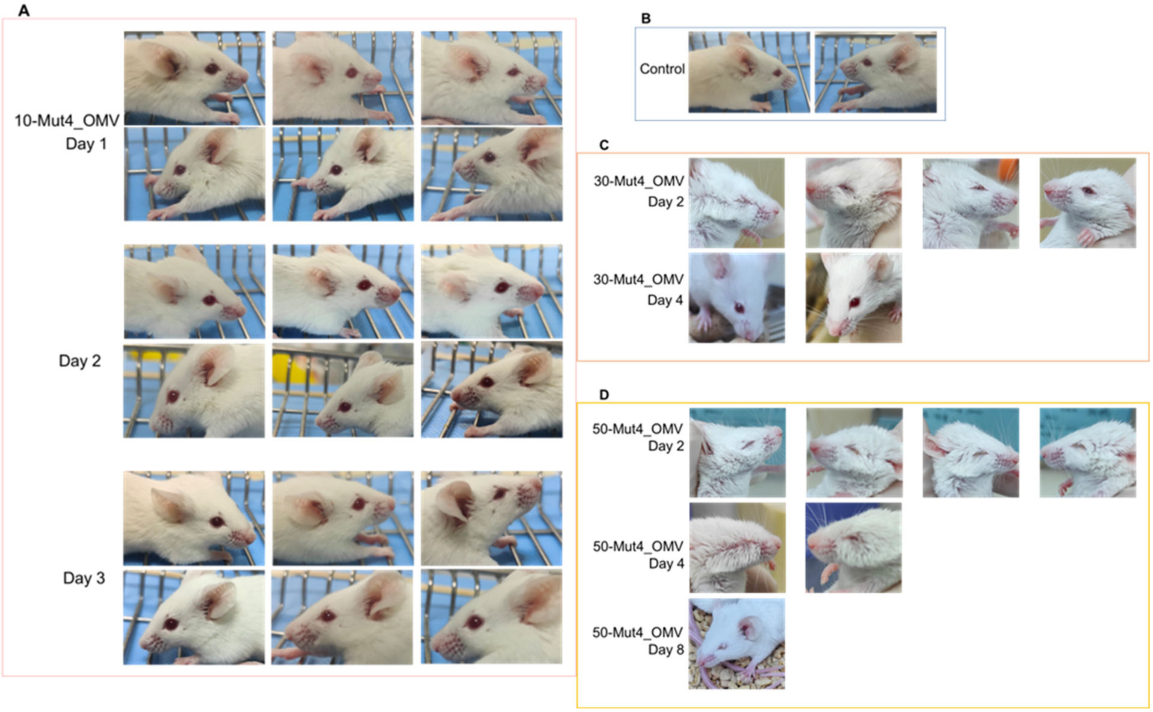

Figure S1. Clinical Status of mice injected with 30μg or 50μg Mut4_OMV. mice in the 10-μg Mut4_OMV cohort exhibited no ocular abnormalities (A) Compare with Control group (B). Mice administered 30 μg of Mut4_OMV demonstrated excessive ocular discharge, piloerection, and lethargic behavior during the initial 3-day observation period, with complete resolution of symptoms by Day 4 (C). In contrast, one animal in the 50-Mut4_OMV cohort (n=5 per group) exhibited severe ocular secretion resulting in complete eyelid closure (D).

.

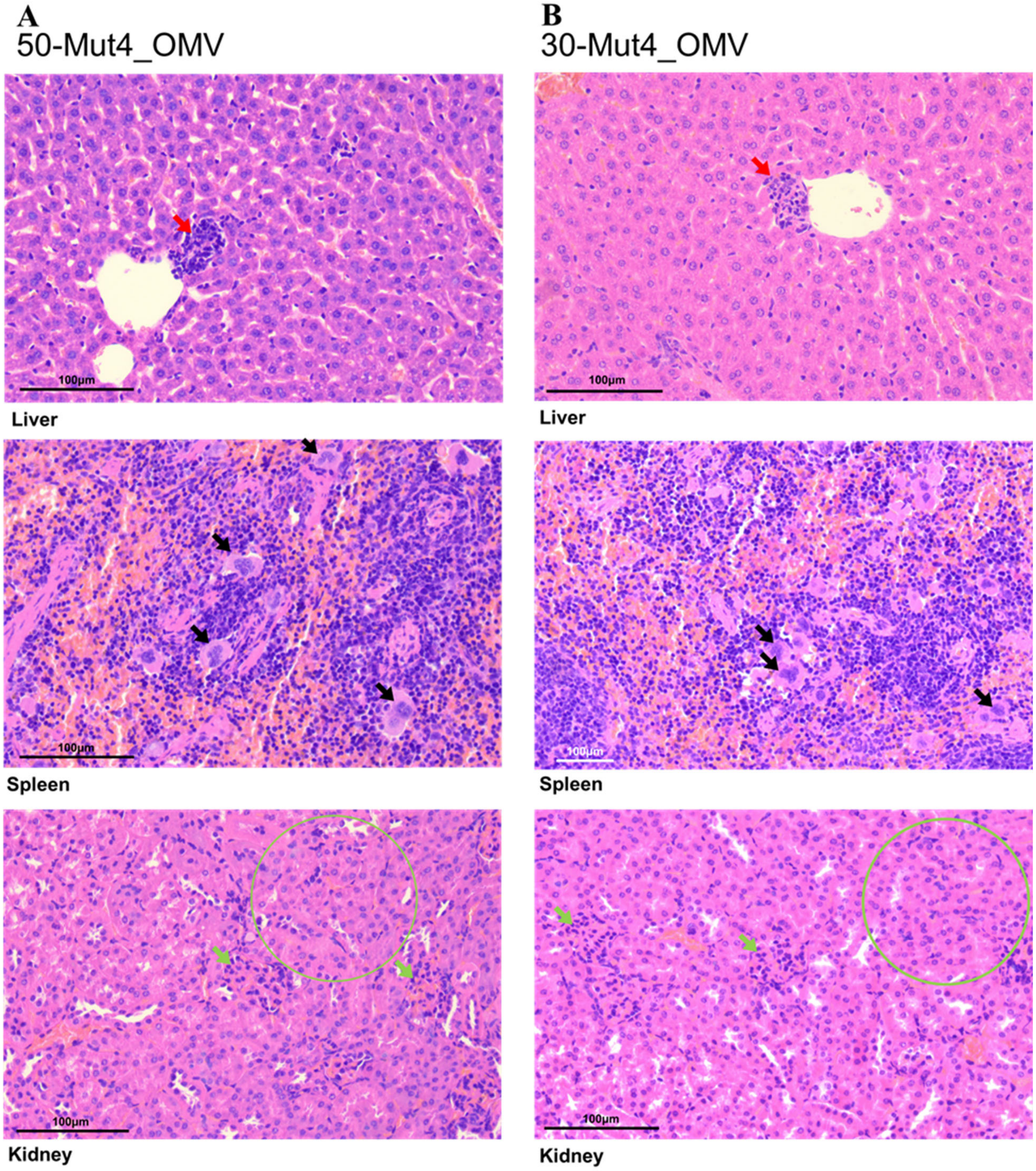

Figure S2. Histopathology of the liver, spleen, and kidney from group 30- and 50-Mut4_OMV mice. Red arrows indicate immune cell infiltration central vein of Hepatic lobules, black arrows indicate Megakaryocytes in red pulp of spleen, green arrows indicate the loss of architecture of glomerulus, and renal tubules (green arrows).
